# Use of expanded carrier screening for retrospective diagnosis of two deceased siblings with Van Maldergem syndrome 2: case report

**DOI:** 10.2478/abm-2022-0036

**Published:** 2023-08-01

**Authors:** Nasim Rahmani, Mohammad Ahmadvand, Golnaz Khakpour

**Affiliations:** Department of Medical Genetics and Molecular Biology, School of Medicine, Iran University of Medical Sciences, Tehran 1449614535, Iran; Department of Oncology and Stem Cell Transplantation, Shariati Hospital, School of Medicine, Tehran University of Medical Sciences, Tehran 1411713135, Iran; Department of Eye Research Center, The Five Senses Institute, Rassoul Akram Hospital, School of Medicine, Iran University of Medical Sciences, Tehran 1445613131, Iran

**Keywords:** carrier screening, exome sequencing, Van Maldergem syndrome, FAT4, congenital anomalies

## Abstract

Van Maldergem syndrome (VMLDS) is a recessive disease which affects multiple organs including the face, ear, and limb extremities. It can be caused by pathogenic variants in either the gene DCHS1 or FAT4. Diagnosis of VMLDS is complicated, especially regarding its similarity of symptoms to Hennekam syndrome, another disorder caused by FAT4 variants. Reported patients are two infantile siblings with multiple congenital anomalies, who deceased without clinical diagnosis. Whole exome sequencing was exploited for expanded carrier screening (ECS) of their parents, which revealed a novel splicing variant in the gene FAT4, NM_024582.6: c.7018+1G>A. In silico analysis of the variant indicates loss of canonical donor splice site of intron 6. This variant is classified as pathogenic based on ACMG criteria. Reverse phenotyping of patients resulted in likely diagnosis of VMLDS2. This study reaffirms the possibility of using ECS, leading to the genetic diagnosis of a rare disease with complicated clinical features.

Van Maldergem syndrome (VMLDS/VMS) and Hennekam lymphangiectasia–lymphedema syndrome (HKLLS/HS) are two rare autosomal recessive disorders with highly similar features which were described around three decades ago. Overlapping characteristics of both syndromes include dysmorphic facial features such as epicanthal folds, blepharophimosis, hypertelorism, flat nasal bridge, microstomia besides to microtia, genital involvement, camptodactyly, syndactyly, and developmental delay. However, there are distinctive signs, presented differentially in each syndrome, as lymphangiectasia and lymphedema, which are associated with HKLLS and abnormal findings in brain magnetic resonance imaging (MRI) such as large anterior fontanelles, neonatal feeding, and breath difficulties, which are associated with VMLDS [1, 2, 3, 4]. Tracheal and renal abnormalities are also common among patients with VMLDS [5].

*FAT4* gene, also known as *FAT–J*, encodes a member of the cadherin protein family in humans and its pathogenic variants were revealed as causality in patients with VMLDS or HKLLS [6, 7]. VMLDS and HKLLS can also be caused by other genes. Variants of *DCHS1* in VMLDS and *CCBE1*, *ADAMTS3* and *FBXL7* in HKLLS, have been identified as well. These syndromes have been numbered according to the related causative genes in the OMIM database (https://www.omim.org/), suggesting that VMLDS2 (MIM number: # 615546) and HKLLS2 (MIM number: # 616006) are caused by the gene *FAT4* [7, 8, 9, 10]. In addition, VMLDS1, caused by DCHS1, represents abnormalities similar to VMLDS2 that may not be clinically distinguishable.

Genetic carrier testing, a method for identifying pathogenic variants in asymptomatic heterozygote parents, emerged in 1970 for hemoglobinopathies. To date, owing to the potency of next-generation sequencing (NGS) methods, it has evolved to expanded carrier screening (ECS), encompassing a broad range of diseases with variable number of genes [11]. Here, we report the usage of whole exome sequencing (WES) for ECS of consanguineous parents with two infants born with multiple congenital anomalies (MCA) and died without exact diagnosis. WES revealed a heterozygous pathogenic variant in the gene *FAT4* followed by revaluation of phenotypic characteristics of the patients, which showed comparability with VMLDS2. Therefore, this finding was considered as the underlying cause of the disease.

## Case report

**Figure 1.**
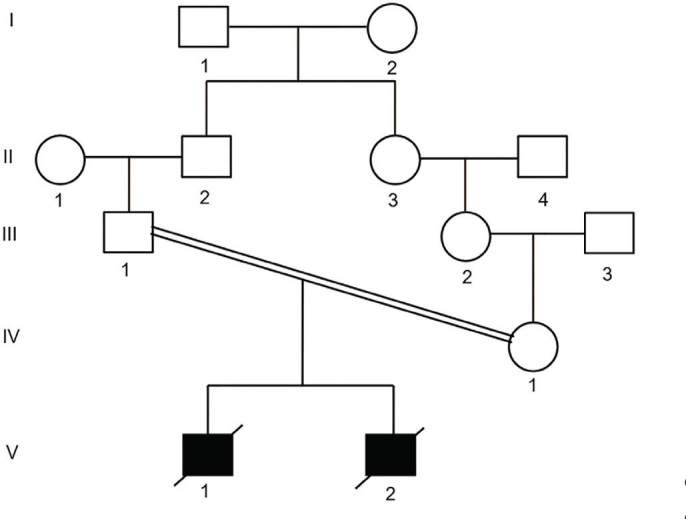
Pedigree of the patients. Proband 1 (V:1) was born from consanguineous first cousins once removed parents (III:1 and IV:1) and was affected by MCA resembling VMS. His sibling, proband 2, (V:2) had a similar phenotype. Both deceased in the second month of life. Circle, female; Square, male; VMS, Van Maldergem syndrome.

They were two male siblings with a similar set of malformations and their asymptomatic parents were first cousins. **Figure 1** shows the pedigree of patients. Regarding evidence indicative to a possible genetic defect, six months after the death of the second child, parents were referred for genetic counseling. Written informed consent form for genetic analysis and publication of clinical and genetic data was obtained from the parents.

All prenatal screening laboratory tests were normal for both patients and they were born via cesarean section.

Proband 1: He was the first child of a healthy 22-year-old mother and a 26-year-old father, with normal prenatal sonography. After his birth, MCA was observed, including tracheomalacia with tachypnea, leading to tracheostomy in the early weeks after birth. He had facial characteristics of prominent eyes, epicanthal folds, micrognathia, microstomia, and bulbous nose together with short neck, low set ears, microtia with aural atresia and conductive hearing impairment, and clubfoot. No more prenatal or neonatal information is available for this patient.

Proband 2: He was the mother's second delivery at age 24. Several minor abnormalities were found in his fetal sonographies: Nuchal translucency (NT) was 3 mm at gestational age of 12 6/7 weeks. Due to this finding, chromosome analysis of amniotic fluid was performed at gestational age of 15 1/7 weeks. It showed a normal male karyotype 46, XY, which was confirmed by his peripheral blood karyotyping after birth. Prenatal ultrasound at gestational age of 17 3/7 weeks showed breech presentation, normal fetal abdomen, and echogenic bowel. At gestational age of 21 weeks, in addition to echogenic bowel, lateral ventricles were found to be at the upper limit of normal range (9.6 mm). No bowel obstruction or brain abnormality could be found by ultrasound imaging. His birth weight was 2500 g with an Apgar score of 9. Similar to proband 1, after birth he had anomalies of prominent eyes, epicanthal folds, micrognathia, microstomia, and bulbous nose with short neck, low set ears, microtia with aural atresia, and conductive hearing impairment, but limb abnormality was in the form of camptodactyly. In addition, minor cardiac abnormalities were present, including patent foramen ovale (PFO) and mild physiologic tricuspid regurgitation (TR) still in normal ranges. Ultrasound sonography revealed two corticomedullary cysts in his right kidney.

Due to tracheostomy of both of them in the early weeks of life, it is not possible to rule out/in feeding difficulties. The only abnormal finding in laboratory tests of proband 1 was a high level of C-reactive protein (CRP) without evidence of infectious disease. They had respiratory distress and were under ventilator for weeks. Both the patients deceased in the second month of life due to hypoxemia and cardiac arrest.

## Method

Carrier screening of parents was carried out using WES. In brief, Twist Human Core Exome Plus Kit (Twist biosciences, San Francisco, CA, USA) was used for exome enrichment and the library was sequenced on Illumina platform (Illumina Inc., San Diego, CA, USA). Mean on-target coverages were 131X and 167X for the mother and father, respectively.

In silico pathogenicity assessments: Allele frequency of the variant was investigated in population databases of 1000genome project (http://www.1000genomes.org), gnomAD (https://gnomad.broadinstitute.org/), ExAC (data available in gnomAD browser), and our local database Iranome (http://www.iranome.ir/). Its damaging effect on gene product was assessed using online prediction tools MutationTaster (http://www.mutationtaster.org/) and Combined Annotation Dependent Depletion (CADD) (https://cadd.gs.washington.edu/) [12, 13]. Web-based splice site prediction tools NNSPLICE v0.9 (https://www.fruitfly.org/seq_tools/splice.html), SD-score (https://www.med.nagoya-u.ac.jp/neurogenetics/SD_Score/sd_score.html) and NetGen2 v2.4 (http://www.cbs.dtu.dk/services/NetGene2/) were applied for further analysis of spliceogenic effects of the variant [14, 15, 16, 17].

This study was in conformity with the Helsinki Declaration and ethical approval was not required for this study in accordance with the local/national guidelines. Written informed consent was obtained from the parent of patients for publication of the details of their medical case.

## Genetic finding

WES data analysis revealed a heterozygous intronic variant, NM_024582.6: c.7018+1G>A, in the gene *FAT4* which was confirmed in parents by sanger sequencing. This variant substitutes the first nucleotide, G, of intron 6 for A, altering canonical splice site (CSS).

The variant was absent from population databases of 1000genome, gnomAD, ExAC, our local database Iranome and, as far as we know, has not been reported before. MutationTaster predicted it as the cause of the disease. In addition, the CADD Phred-score (GRCh37-v1.6 adapted especially for splicing variants) is 33 for this variant, which puts it among the top 0.1% most deleterious substitutions that happen throughout the human genome. We applied splice site prediction tools to analyze the mutant sequence and detect possible cryptic donor sites. All analyses indicated that the CSS is missed and suggested new donor sites; **Table 1** shows in silico scores of the normal and mutant CSS.

**Table 1.** Comparison of normal CSS, GT, in the intron 6 of *FAT4* and its mutant, AT, by in silico splice prediction tools and two predicted cryptic donor sites

	**Sequences exon/intron**	**Position in intron 6**	**NNSPLICE**	**NetGene2**	**SD-score**
Normal CSS	GATTCAG/**GT**AAGTCC	+1	1.00	1–0.83	−2.074
Mutant CSS	GATTCAG/**AT**AAGTCC	+1	-	-	-
Predicted donor sites	CATGGTG/**GT**GCGTGC	+191	0.74	0–0.41	−3.998
	GACATGG/**GT**GAGTGA	+683	0.99	1–0.00	−2.277

CSS, canonical splice site.

Based on criteria from the ACMG guideline, this variant is classified as pathogenic [18, 19]. Applied rules include PVS1 for being a null variant in a gene for which loss of function is the mechanism of disease and PM2 for the variant is not found in the population databases.

In addition to *FAT4*, the parents shared heterozygous variants of other genes, which are present in **Table 2**.

**Table 2.** Table shows variants detected in three genes that parents are heterozygous carriers for them. They share common variants in *PINK1* and *PTPRQ* genes, but they are carriers for different variants in *MYO15A* gene

**Gene/transcript**	**Variant**	**Loc.**	**Chr. pos.**	**Related phenotypes**	**OMIM number**	**Inh.**	**Class^†^**
**The couple both are heterozygous carriers for the same variants in *PINK1* and *PTPRQ* genes**							
***PINK1*** NM_032409	c.709A>G p.M237V	Exon 3	Chr1: 20966418	Early onset Parkinson disease 6	605909	AR	VUS
***PTPRQ‡*** NM_001145026	c.3446-5 dupT	Intron 21	Chr12: 80542081	Autosomal recessive deafness 84A	613391	AR	Benign
				Autosomal dominant deafness-73	617663	AD	
**The couple are heterozygous carriers for different variants in *MYO15A* gene**							
***MYO15A*** NM_016239	c.3622C>T p.R1208C	Exon 3	Chr17: 18027809	Autosomal recessive deafness-3	600316	AR	VUS
	c.5230T>A P.S1744T	Exon 20	Chr17: 18043849				VUS

† Variants classification based on ACMG standards and guidelines for the interpretation of sequence variants, 2015.

‡ Exceptionally chromosomal position of PTPRQ gene is presented based on GRCh38 assembly because it has been presented this way in related databases.

Chr. Pos., Chromosomal position; Inh, Inheritance pattern; Loc., Location; VUS, variants with uncertain significance; ACMG, American College of Medical Genetics and Genomics; AD, autosomal dominant; AR, autosomal recessive; CNV, copy number variation; EGF, epidermal growth factor; GRCh38, Genome Reference Consortium Human Build 38; OMIM, Online Mendelian Inheritance in Man; PGD, Preimplantation Genetic Diagnosis; PND, Prenatal diagnosis.

## Discussion

We report two siblings with MCA, born to consanguineous parents, who deceased at the age of two months. During genetic counseling, their parents were explained about a possible familial recessive disorder and informed that in the absence of an accurate clinical diagnosis, a genotype-first diagnosis may be achieved. Therefore, WES was suggested and, due to no samples from their deceased children, it was applied for carrier screening of the parents. Also, they were informed about the advantages and disadvantages of this method. The advantages of WES in ECS, even in cases with no post-mortem samples, have been approved through previous studies. For example, in a recently published report, by this method they could identify the causal variant of a metabolic disorder in three dead siblings. However, as far as we know, in all of them there have been a definite or probable clinical diagnosis. In the current study, no preliminary clinical diagnosis or assumption were given to deceased patients, and from this point of view it could be assumed as the first report of its kind [20, 21, 22].

This approach led to identification of a number of variants shared with the parents, including a novel pathogenic heterozygous variant in the gene *FAT4*, NM_024582.6: c.7018+1G>A. (Please see **Table 2** for the other variants). This variant is pathogenic by applied rules PVS1 and PM2. Regarding criteria PVS1, loss of function is an established pathogenicity underlying the diseases associated with *FAT4* variants: (1) According to data from Simple ClinVar database (https://simple-clinvar.broadinstitute.org/), most of the pathogenic or likely pathogenic variants in the gene *FAT4* are of types which have disrupting consequence on protein function, including stopgain, frameshift, and splicing variants; (2) the OMIM database indicates several animal studies which support the idea of loss of function in *FAT4*-related disorders [23].

Reverse phenotyping of the patients showed overlapping features of syndromes caused by *FAT4* variants, HKLLS, and VMLDS, but the absence of lymphangiectasia or lymphedema, which are hallmarks of HKLLS, and the presence of tracheomalacia in both the siblings with small cysts in the kidney of proband 2 can be suggestive of VMLDS2 [1]. **Table 3** shows a comparison of the clinical symptoms between the patients of this study with characteristics previously described in the literature. As mentioned before, clinical variable expressivity is common across patients with *FAT4* variants, which makes the classification of patients in Van Maldergem or Hennekam syndrome challenging. An interesting case is a patient, F4–1, from Alders et al.'s [6] study, who had a splicing variant, NM_001291303.1: c.7200–2A>C, disrupting the acceptor site of intron 8. They showed by functional study that it leads to deletion of only two amino acids. Comparing the clinical symptoms of this patient with our patients shows significant clinical differences. She, F4–1, has been consistent with Hennekam syndrome, due to having the lymphatic anomalies with no tracheal or kidney abnormalities. Of course, overlapping symptoms are also seen in these three patients, such as dysmorphic faces and skeletal limb anomalies, evident in the form of camptodactyly in our patients, but in the form of syndactyly in the previous patient. She, F4–1, had also ear anomalies but without hearing problems. It is noteworthy that this pediatric patient, F4–1, has been alive at the time of report.

**Table 3.** Phenotypic comparison of P1 and P2 with HKLLS and VMLDS characteristics previously described in the literature [1, 4, 5, 24]

**Abnormalities**	**HS**	**VMS**	**P1**	**P2**
Microcephaly	+	++	No	No
Large fontanelle	−	+++	No	No
Blepharo-nasal malformation	+++	+++	Yes	Yes
Micrognathia and small mouth	+++	+++	Yes	Yes
Irregular dentition	+++	+++	NA	NA
Short stature	+++	+++	NA	NA
Hypertelorism	+++	+++	No	No
Epicanthic folds	+++	+++	Yes	Yes
Camptodactyly	++	+++	No	Yes
Syndactyly	+	+	No	No
Clubfoot	+	+	Yes	No
Microtia	+++	+++	Yes	Yes
Conductive hearing loss	+*	+++	Yes	Yes
Cardiac malformation	+*	+	No	Yes
Infantile hypotonia	+*	+++	No	No
Developmental delay	+++	+++	NA	NA
Feeding difficulties	+	+++	Yes	Yes
Choanal atresia/stenosis	−	++	No	No
Tracheal anomalies	−	+++	Yes	Yes
Periventricular nodular heterotopia	−	++	Not performed	
Corpus callosum anomalies	−	++	Not performed	
Other brain anomalies	+*	+		
Renal anomalies	−	+++	No	Yes
Genital anomalies	edema	+	No	No
Lymphedema limb	+++	+*	No	No
Primary intestinal lymphangiectasia	+++	−*	No	No
Other lymphangiectasia	++	−	No	No

+++, common.

++, around a half of patients.

+, less than half of patients.

+*, a few patients.

−*, only one exception.

HKLLS, Hennekam lymphangiectasia-lymphedema syndrome; VMS: Van Maldergem syndrome; P1, proband 1; P2, proband 2; NA, not applicable.

The Fat cadherin subfamily are highly conserved single-pass transmembrane receptors, which in humans consist of four members FAT1–4. All FAT cadherins have a large extra-cellular domain mainly consisting of cadherin repeats, which are followed by a number of EGF-like and Laminin G-like motifs [25]. Heterophilic interaction of FAT4 with DCHS1, another cadherin, is critical in planar cell polarity (PCP) and Hippo signaling pathways involved in cellular proliferation, orientation, migration, and tissue development [26].

**Figure 2.**
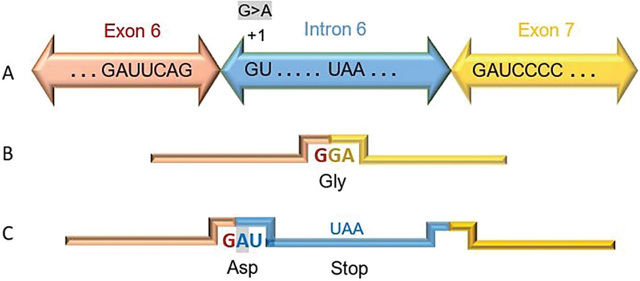
Possible consequence of the variant c.7018+1G>A, NM_024582.6, on the transcript. **(A)** Normal splicing, using 5’ CSS, joins exon 6 to 7 and **(B)** the junction forms Glycine codon (GGA). **(C)** This variant can result in the loss of 5′ CSS and aberrant splicing. Theoretically, insertion of the fragment from intron 6 in the transcript creates the codon of Aspartic acid in junction and leads to frameshift creating a premature stop codon, UAA, in position +33 to +35. CSS, canonical splice site; nts, nucleotides,

*FAT atypical cadherin 4*, *FAT4*, is located on chromosome 4q28.1 and its transcript, NM_024582.6, contains 18 exons which is translated to a protein with 4981 amino acids [27]. The novel pathogenic splicing variant, c.7018+1 G>A, disturbs 5′ CSS at donor sequence motif of intron 6 (**Figure 2**). In silico analysis of mutant sequence using splice prediction tools showed that at least two cryptic splice sites inside intron 6 can be potentially activated, of which one of them is very strong (**Table 1**). Although clinical evidence supports our idea of abnormal *FAT4* transcription with no functional product, unfortunately DNA or tissue samples from patients are not available, and due to remote location of the parents it is not possible to conduct further RNA studies.

Diagnosis of rare diseases with variable expressivity and overlapping features just based on clinical evaluations is difficult, especially in infantile patients. NGS, as a powerful facility, can unravel genetic defects underlying these group of disorders. Moreover, reverse phenotyping after a genetic finding may extend the clinical assessments and anticipate diagnosis of late onset features [28]. Besides these advantages, NGS has ECS test to hundreds of conditions that makes it increasingly favorable for preconception, preimplantation, or prenatal diagnostic procedures (PND or PGD) [29].

However, next to these advantages, there are routine limitations associated with NGS methods, including the lack of full coverage for all genes or exons, absence of deep intronic regions in WES, and inability to investigate CNVs or chromosomal rearrangements. Moreover, finding variants with uncertain significance (VUS) can bring even more complexity to the situation. Furthermore, there are additional challenges for cases similar to ours: first, the heterozygous variant is identified in healthy adults and cannot be confirmed in a homozygous patient. Second, genotype–phenotype correlation is not straightforward and there is always concern around other possibilities which may be missed. This makes genetic counseling complicated, and any clinical intervention based on these findings must be advised more cautiously [22, 30]

## Conclusion

ECS of parents is a powerful test which can reveal the genetic defect in deceased patients suspected to have a rare disease with recessive inheritance pattern. Here, exploiting this method revealed a novel pathogenic splice variant in *FAT4* gene and resulted in diagnosis of VMLDS in two dead infantile siblings.

## References

[1] Ivanovski I, Akbaroghli S, Pollazzon M, Gelmini C, Caraffi SG, Mansouri M (2018). Van Maldergem syndrome and Hennekam syndrome: further delineation of allelic phenotypes. Am J Med Genet A..

[2] Hennekam RC, Geerdink RA, Hamel BC, Hennekam FA, Kraus P, Rammeloo JA, Tillemans AA (1989). Autosomal recessive intestinal lymphangiectasia and lymphedema, with facial anomalies and mental retardation. Am J Med Genet..

[3] van Maldergem L, Wetzburger C, Verloes A, Fourneau C, Gillerot Y (1992). Mental retardation with blepharo-naso-facial abnormalities and hand malformations: a new syndrome?. Clin Genet..

[4] Musumeci ML, Nasca MR, De Pasquale R, Schwartz RA, Micali G (2006). Cutaneous manifestations and massive genital involvement in Hennekam syndrome. Pediatr Dermatol..

[5] Mansour S, Swinkels M, Terhal PA, Wilson LC, Rich P, Van Maldergem L (2012). Van Maldergem syndrome: further characterisation and evidence for neuronal migration abnormalities and autosomal recessive inheritance. Eur J Hum Genet..

[6] Alders M, Al-Gazali L, Cordeiro I, Dallapiccola B, Garavelli L, Tuysuz B (2014). Hennekam syndrome can be caused by FAT4 mutations and be allelic to Van Maldergem syndrome. Hum Genet..

[7] Cappello S, Gray MJ, Badouel C, Lange S, Einsiedler M, Srour M (2013). Mutations in genes encoding the cadherin receptor-ligand pair DCHS1 and FAT4 disrupt cerebral cortical development. Nat Genet..

[8] Alders M, Hogan BM, Gjini E, Salehi F, Al-Gazali L, Hennekam EA (2009). Mutations in CCBE1 cause generalized lymph vessel dysplasia in humans. Nat Genet..

[9] Brouillard P, Dupont L, Helaers R, Coulie R, Tiller GE, Peeden J (2017). Loss of ADAMTS3 activity causes Hennekam lymphangiectasia-lymphedema syndrome 3. Hum Mol Genet..

[10] Boone PM, Paterson S, Mohajeri K, Zhu W, Genetti CA, Tai DJC (2020). Biallelic mutation of FBXL7 suggests a novel form of Hennekam syndrome. Am J Med Genet A..

[11] Sparks TN (2020). Expanded carrier screening: counseling and considerations. Hum Genet..

[12] Rentzsch P, Witten D, Cooper GM, Shendure J, Kircher M (2019). CADD: predicting the deleteriousness of variants throughout the human genome. Nucleic Acids Res..

[13] Schwarz JM, Cooper DN, Schuelke M, Seelow D (2014). MutationTaster2: mutation prediction for the deep-sequencing age. Nat Methods..

[14] Celniker SE, Rubin GM (2003). The *Drosophila melanogaster* genome. Annu Rev Genomics Hum Genet..

[15] Sahashi K, Masuda A, Matsuura T, Shinmi J, Zhang Z, Takeshima Y (2007). In vitro and in silico analysis reveals an efficient algorithm to predict the splicing consequences of mutations at the 5' splice sites. Nucleic Acids Res..

[16] Hebsgaard SM, Korning PG, Tolstrup N, Engelbrecht J, Rouzé P, Brunak S (1996). Splice site prediction in Arabidopsis thaliana pre-mRNA by combining local and global sequence information. Nucleic Acids Res..

[17] Brunak S, Engelbrecht J, Knudsen S (1991). Prediction of human mRNA donor and acceptor sites from the DNA sequence. J Mol Biol..

[18] Richards S, Aziz N, Bale S, Bick D, Das S, Gastier-Foster J (2015). Standards and guidelines for the interpretation of sequence variants: a joint consensus recommendation of the American College of Medical Genetics and Genomics and the Association for Molecular Pathology. Genet Med..

[19] Kopanos C, Tsiolkas V, Kouris A, Chapple CE, Albarca Aguilera M, Meyer R, Massouras A (2019). VarSome: the human genomic variant search engine. Bioinformatics..

[20] Ellard S, Kivuva E, Turnpenny P, Stals K, Johnson M, Xie W (2015). An exome sequencing strategy to diagnose lethal autosomal recessive disorders. Eur J Hum Genet..

[21] Sallevelt SCEH, de Koning B, Szklarczyk R, Paulussen ADC, de Die-Smulders CEM, Smeets HJM (2017). A comprehensive strategy for exome-based preconception carrier screening. Genet Med..

[22] Tran VK, Diep QM, Qiu Z, Le TP, Do LD, Tran HA (2022). Whole exome sequencing analysis in a couple with three children who died prematurely due to carnitine-acylcarnitine translocase deficiency. Taiwan J Obstet Gynecol..

[23] (2022). FAT ATYPICAL CADHERIN 4; FAT4 [Internet].

[24] de Meij TGJ, Zwijnenburg PJG, Broers CJM, Bökenkamp A (2019). Intestinal lymphangiectasia—a novel finding in Van Maldergem syndrome challenging the role of lymphedema for the distinction from Hennekam syndrome. Am J Med Genet A..

[25] Sadeqzadeh E, de Bock CE, Thorne RF (2014). Sleeping giants: emerging roles for the fat cadherins in health and disease. Med Res Rev..

[26] Blair S, McNeill H (2018). Big roles for Fat cadherins. Curr Opin Cell Biol..

[27] Yates AD, Achuthan P, Akanni W, Allen J, Allen J, Alvarez-Jarreta J (2022). ENSEMBL release 101 [Internet].

[28] Fernandez-Marmiesse A, Gouveia S, Couce ML (2018). NGS technologies as a turning point in rare disease research, diagnosis and treatment. Curr Med Chem..

[29] Kraft SA, Duenas D, Wilfond BS, Goddard KAB (2019). The evolving landscape of expanded carrier screening: challenges and opportunities. Genet Med..

[30] Komlosi K, Diederich S, Fend-Guella DL, Bartsch O, Winter J, Zechner U (2018). Targeted next-generation sequencing analysis in couples at increased risk for autosomal recessive disorders. Orphanet J Rare Dis..

